# Petasin and isopetasin reduce CGRP release from trigeminal afferents indicating an inhibitory effect on TRPA1 and TRPV1 receptor channels

**DOI:** 10.1186/s10194-021-01235-5

**Published:** 2021-04-13

**Authors:** Johanna Kleeberg-Hartmann, Birgit Vogler, Karl Messlinger

**Affiliations:** Institute of Physiology and Pathophysiology, Friedrich-Alexander-University of Erlangen-Nürnberg, Universitätsstraße 17, 91054 Erlangen, Germany

**Keywords:** Petasin, Isopetasin, Butterbur, CGRP, TRPA1, TRPV1, Migraine

## Abstract

**Background:**

Butterbur root extract with its active ingredients petasin and isopetasin has been used in the prophylactic treatment of migraine for years, while its sites of action are not completely clear. Calcitonin gene-related peptide (CGRP) is known as a biomarker and promoting factor of migraine. We set out to investigate the impact of petasins on the CGRP release from trigeminal afferents induced by activation of the calcium conducting transient receptor potential channels (TRPs) of the subtypes TRPA1 and TRPV1.

**Methods:**

We used well-established in vitro preparations, the hemisected rodent skull and dissected trigeminal ganglia, to examine the CGRP release from rat and mouse cranial dura mater and trigeminal ganglion neurons, respectively, after pre-incubation with petasin and isopetasin. Mustard oil and capsaicin were used to stimulate TRPA1 and TRPV1 receptor channels. CGRP concentrations were measured with a CGRP enzyme immunoassay.

**Results:**

Pre-incubation with either petasin or isopetasin reduced mustard oil- and capsaicin-evoked CGRP release compared to vehicle in an approximately dose-dependent manner. These results were validated by additional experiments with mice expressing functionally deleted TRPA1 or TRPV1 receptor channels.

**Conclusions:**

Earlier findings of TRPA1 receptor channels being involved in the site of action of petasin and isopetasin are confirmed. Furthermore, we suggest an important inhibitory effect on TRPV1 receptor channels and assume a cooperative action between the two TRP receptors. These mechanisms may contribute to the migraine prophylactic effect of petasins.

## Background

### Migraine and the trigeminovascular system

Migraine is a complex neurological disorder characterized by a commonly unilateral, frequently severe headache, possibly preceded by an aura and accompanied by sensory and autonomic dysfunctions like photo- and phonophobia, nausea and vomiting [1–3]. While the neurological and autonomic symptoms are thought to be mainly caused by central disturbances affecting also the central nociceptive pathways, the migraine headache is believed to depend fundamentally on the activation and sensitization of the trigeminovascular system, which consists of trigeminal afferents innervating intracranial arterial blood vessels [4–7]. Activation of trigeminal afferents, in particular meningeal afferents innervating the dura mater, is very likely responsible for the activation of central pain pathways and finally for the generation of migraine pain [8, 9].

### Role for TRP receptors in trigeminal nociception

Transient receptor potential (TRP) channels play an important role in the transduction of cell signals and thus also in trigeminal nociception. Transient receptor potential vanilloid type 1 (TRPV1) receptor channels detect heat and are activated or sensitized by chemical stimuli (e.g. capsaicin and extracellular protons). TRPV1 receptor channels are expressed by the majority of nociceptive trigeminal afferents, mostly C-fiber but also a minority of Aδ fiber afferents. Interestingly, 70% of CGRP-immunoreactive neurons were found to be colocalized with TRPV1 channels [10, 11].

Transient receptor potential ankyrin type 1 (TRPA1) receptor channels are nearly exclusively expressed by C-fibers. They are activated by multiple exogenous chemical agents (e.g. isothiocyanates, acrolein and formalin) and endogenous inflammatory stimuli (e.g. bradykinin). Many TRPA1-positive neurons co-express TRPV1 channels and can be sensitized or activated by an increase in intracellular calcium, i.e. also by activation of calcium conducting TRPV1 receptor channels [12–14]. Some experimental evidence exists for either a cooperative effect between TRPV1 and TRPA1 receptor channels [15], an TRPV1-inhibiting effect of TRPA1 [16], or an ambiguous role of the TRPA1 channel as both an activator [17] and an inhibitor of spinal trigeminal neurons [18].

### What is the link of petasins and CGRP in migraine?

Due to the high prevalence and the severe symptom burden, the pathophysiology and therapy of migraine have been subject of research for decades [3, 6, 8, 19]. An effective phytotherapeutical option in migraine prophylaxis is an extract from the rhizome of the plant butterbur (*Petasites hybridus*). Butterbur root extract with its active ingredients petasin and isopetasin (petasins) has been used in migraine prophylaxis for many years [20–22]. However, the site of action is still not clear. Among the different effects that are attributed to petasins in the trigeminovascular system, their presumed inhibitory effect on the release of neuropeptides, especially calcitonin gene-related peptide (CGRP), is of particular relevance. CGRP is released from activated trigeminal afferents. Trigeminal afferent nerve fibers of the cranial dura mater [23], of pial and intracerebral blood vessels [24] and of the trigeminal ganglion [25, 26] are peripheral sites of CGRP release, while the central source of CGRP are fibers of the spinal trigeminal nucleus [27]. The highest concentration of CGRP is found in the trigeminal ganglion [28] in which 48% of the neurons have been found CGRP-immunopositive [26]. Upon activation of primary afferents, CGRP is released by Ca^2+^-dependent exocytotic mechanisms [29]. When released in pathological concentrations, CGRP can likely act as nociceptive mediator possibly contributing to the initiation and continuance of nociceptive events [30, 31].

Previous preclinical experiments have shown that CGRP release from central afferent terminals in a mouse spinal cord in vitro preparation is reduced by isopetasin [32]. Also, CGRP secretion from neuroendocrine CA77 cells was reduced by 1 μM S-petasin, a constituent of butterbur extract, which inhibited also calcium influx [33]. Therefore, it is of considerable interest to know if and by which mechanisms petasins may be able to reduce the stimulated CGRP release from trigeminal afferents innervating the trigeminovascular system, particularly the cranial dura mater.

### Petasins – a therapeutic option in migraine prophylaxis, but how does it work?

Common butterbur (*Petasites hybridus*), domestic in Europe and north western Asia, belongs to the Asteracae and has been used for centuries in folk medicine for the treatment of many different symptoms like spastic pain, dysmenorrhea, cough, and wound healing [34]. Since the 1950s, medical interest in butterbur has newly grown. Its spasmolytic effects in the gastrointestinal [35] and the urinary tract [36], and anti-inflammatory activity in allergic rhinitis and asthma have been subjects of medical research [37–39]. A further therapeutic option for the use of the extract from the plant’s rhizome is prophylactic migraine treatment [20–22].

The extract contains several compounds, of which two active agents, the sesquiterpenes petasin and isopetasin, are considered effective in the prevention of migraine pain [40]. Petasin is not stable but converts to its isomer isopetasin spontaneously. Therefore, it is not clear which of the components contributes to which degree to the therapeutic effects [32]. Although butterbur root extract has been used successfully in prophylactic migraine therapy for several years, little is known about the site and mode of action of the petasins. Thomet et al. [41] discussed antiinflammatory effects through inhibition of leukotriene synthesis and the activity of eosinophils, while Wang et al. [42–44] hypothesized that petasins act as a direct antagonist of voltage-gated Ca^2+^-channels (VGCCs) in vascular smooth muscle cells. Blockade of VGCCs could also be found by Horak et al. [45]. Ko et al. [46], too, confirmed the impact of petasins on VGCCs and furthermore supposed that the relaxing effect of petasins is also due to an antimuscarinic action. However, the relevance of these mechanisms for migraine therapy is doubtful. Benemei et al. [32] using calcium imaging and patch clamp recordings found that isopetasin activates TRPA1 channels leading to excitation of neuropeptide containing nociceptors, which finally results in heterologous desensitization and reduced neurogenic inflammation. They proposed that this mechanism may account for the anti-migraine action of butterbur.

Our hypothesis underlying the present preclinical experiments was that petasins and isopetasin reduce the stimulated CGRP release from meningeal afferents and trigeminal ganglia, which may indicate desensitization of TRPV1 or TRPA1 receptor channels.

## Methods

All experiments and procedures were carried out according to the German guidelines and regulations of the care and treatment of laboratory animals and the European Communities Council Directive of November 24, 1986 (86/609/EEC), amended September 22, 2010 (2010/63/EU).

For CGRP release experiments from the dura mater and trigeminal ganglia, home bred adult Wistar rats of both sexes (260–410 g) as well as adult C57 BL/6 mice of both sexes and different genetic configuration were used: C57 BL/6 wild type mice (18–31 g), C57 BL/6 knock out mice with functionally deleted TRPA1 (18–30 g) or deleted TRPV1 (19–31 g) receptor channels. Animals were euthanized by CO_2_ inhalation and decapitated. Rat skulls were freed from skin, muscles, eyes and mandibles, and sagittally divided into two halves. The brain was carefully removed taking care not to damage the dura mater lining the skull. Trigeminal ganglia (TGs) were dissected together with their dura sheath by cutting the trigeminal branches, the ophthalmic, maxillary and mandibular nerves at their utmost distal sites accessible, and kept in wells of a microplate filled with 120 μL of synthetic interstitial fluid (SIF). The microplate was placed on the surface of a pre-warmed water bath (37 °C). Mice skulls were flayed and sagittally divided into two halves. Mice brains were carefully removed from the skull. One of the mice skull halves of each animal was used for dura release experiments, while from the other half the TG was dissected and processed like rat TGs. Skull halves for dura experiments were washed in gently flowing SIF for 30 min at room temperature and subsequently mounted upon the surface of a water bath of 37 °C with the opening upside. Mice skull halves were sealed with a rim of Vaseline® on their outer surface in order to avoid spillage due to hairline cracks in the filigree mouse skull, and mounted accordingly. Skull cavities were filled with 500 μL (rat) or 200 μL (mouse) of SIF. The same volumes were used for pre-incubating solutions for 1 h as well as for the respective incubating steps of 5 min each during CGRP release experiments. Evaporation during pre-incubation was prevented by a self-adhesive plastic film attached on top of the water bath containers.

All tissue samples were incubated with either butterbur root extract solved in ethanol containing different concentrations of petasins (3 μg/mL, 10 μg/mL, 30 μg/mL, 100 μg/mL), isopetasin solved in ethanol (3 μg/mL, 10 μg/mL, 30 μg/mL) or ethanol equivalent to the respective concentration of petasins or isopetasin (0.0316%, 0.105%, 0.316%, 1.055% ethanol) for 1 h and subsequently washed with SIF three times, before fluid samples were sampled for measurements of CGRP concentration using a pipette without touching the tissues. The first two samples were taken after incubation with SIF for 5 min each in order to measure the basal CGRP release from the dura mater and the trigeminal ganglia. Then the TRPA1 receptor agonist allylisothiocyanate (mustard oil, MO) 5 × 10^− 4^ M (Wistar, C57 BL/6 wild type, C57 BL/6 TRPA1 knock-out) or the TRPV1 receptor agonist capsaicin (Caps) 5 × 10^− 7^ M in SIF (C57 BL/6 TRPV1 knock-out) was applied and incubated for 5 min, followed by one 5 min application of SIF. Finally, caps 5 × 10^− 7^ M in SIF (Wistar, C57 BL/6 wild type, C57 BL/6 TRPA1 knock-out), or MO 5 × 10^− 4^ M (C57 BL/6 TRPV1 knock-out) was applied for incubation of 5 min. After each incubation step the fluid was carefully removed and SIF was added for washing the tissues, before the next solution was added. From each sample 100 μl were collected in Eppendorf cups, diluted with 25 μL EIA buffer (Bertin Pharma, France) containing peptidase inhibitors, and stored on ice or deep-frozen until they were processed for CGRP measurement.

CGRP was measured using a CGRP enzyme immunoassay (EIA) kit (Bertin Pharma/SPIbio, Montigny Le Bretonneux, France) according to the manufacturer’s instruction. The sandwich technique of this assay consists of immune reactions of capture and tracer antibodies recognizing different CGRP epitopes, and the enzymatic activity of acetylcholinesterase (AChE, Ellman’s reagent). The emitted light is subsequently spectrophotometrically detected as a measure for the CGRP concentration in the samples, displayed as pg/mL. The detection limit of the assay is 5 pg/mL according to the manufacturer’s information.

Statistical analysis was performed with STATISTICA (StatSoft 7.0, Tulsa, OK). The consecutive measurements at intervals of 5 min were analysed with repeated measures ANOVA and least square difference (LSD) post-hoc test on the basis of the raw data. Comparisons between different concentrations and groups were analysed by univariate ANOVA and least square difference (LSD) post-hoc test on the basis of data normalized to the mean of the two initial CGRP measurements after SIF application in each experiment to compensate for inter-assay variations. Data are presented as means ± SEM, *p* < 0.05 was considered statistically significant. Panels were created with Origin® 2019 (OriginLab, Massachusetts).

Chemicals: Synthetic interstitial fluid (SIF) is composed of (in mM): 107.8 NaCl, 26.2 NaHCO_3_, 9.64 Na-gluconate, 7.6 sucrose, 5.55 glucose, 3.5 KCl, 1.67 NaH_2_PO_4_ × 2 H_2_O 1.53 CaCl_2_ × 2 H_2_O and 0.69 MgSO_4_ × 7 H_2_O, adjusted to pH 7.4. Butterbur root extract (Petadolex®, Weber & Weber, Inning/Ammersee, Germany) was dissolved in ethanol at a concentration of 9.483 mg/mL and step-wise diluted with SIF to the applied concentrations of 100, 30, 10 and 3 μg/mL. Isopetasin (HWI Group, Rülzheim, Germany) was correspondingly dissolved in ethanol and diluted with SIF to the respective concentrations. Accordingly, ethanol concentrations in the vehicle solutions tested were 10.55, 3.16, 1.05 and 0.315 μL/mL in SIF. Mustard oil (MO) (Merck, Darmstadt, Germany) and capsaicin (Caps) (Sigma-Aldrich, Taufkirchen, Germany) were dissolved in ethanol 1% and diluted with SIF to the final concentrations of 5 × 10^− 4^ M (MO) and 5 × 10^− 7^ M (Caps).

## Results

### Butterbur root extract, Wistar

The effect of pre-incubation with butterbur root extract solved in ethanol was tested in the range of 3–100 μg/mL (concentration of petasins in the extract: 3 μg/mL, 10 μg/mL, 30 μg/mL, 100 μg/mL) and also compared to pre-incubation with the solvent ethanol in the respective concentrations (0.0316%, 0.105%, 0.316%, 1.055% ethanol). Raw data was used for repeated measures analysis, while normalized data was used for comparison between ethanol and petasins.

### Dura mater, basal CGRP release

After 1 h treatment of the hemisected skulls with different concentrations of the vehicle (SIF) containing ethanol (0.0316–1.055%), the CGRP concentration in the initial SIF solutions (basal release) varied between 4.4 and 25.3 pg/mL, which is mainly due to the inter-assay variation of the EIA kits. However, since there was no correlation of the basal release with the ethanol concentrations, and the blank values of SIF (6.7–10.3 pg/mL) were also in this range, there was no reason to assume that ethanol at these concentrations caused CGRP release itself. Therefore, the release values of all vehicle experiments (*n* = 12) were averaged and compared with the release values following 1 h treatment with different concentrations of petasins (3–100 μg/mL). There was also no evidence for a CGRP release stimulating effect of the petasins or isopetasin; basal values after SIF varied between 12.7 and 21.3 pg/mL without any correlation to the doses used; blank values were 8.5–10.8 pg/mL. Raw data with deviations are displayed in Tables 1, 2, 3.

**Table 1:**
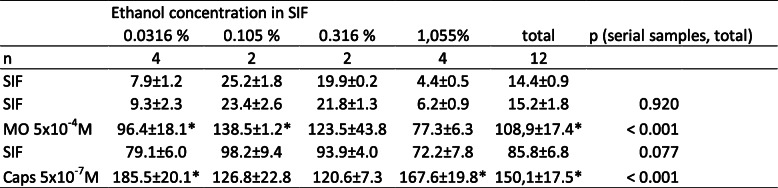
Dura mater encephali, different ethanol concentrations. CGRP release in pg/ml, all values are means ± SEM, *p < 0.05

**Table 2:**
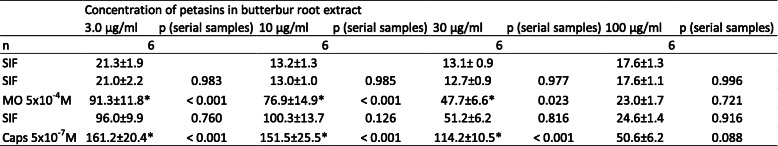
Dura mater encephali, different petasin concentrations. CGRP release in pg/ml, all values are means ± SEM, *p < 0.05

**Table 3:**
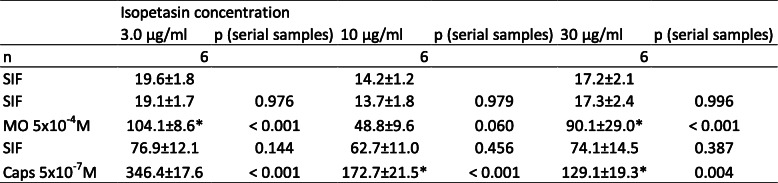
Dura mater encephali, different isopetasin concentrations. CGRP release in pg/ml, all values are means ± SEM, *p < 0.05

For all concentrations of petasins in butterbur root extract and ethanol without extract, the basal CGRP release from the dura mater encephali within 5 min was 15.5 ± 1.3 pg/mL (*n* = 36).

### Dura mater, stimulated CGRP release

**Fig. 1:**
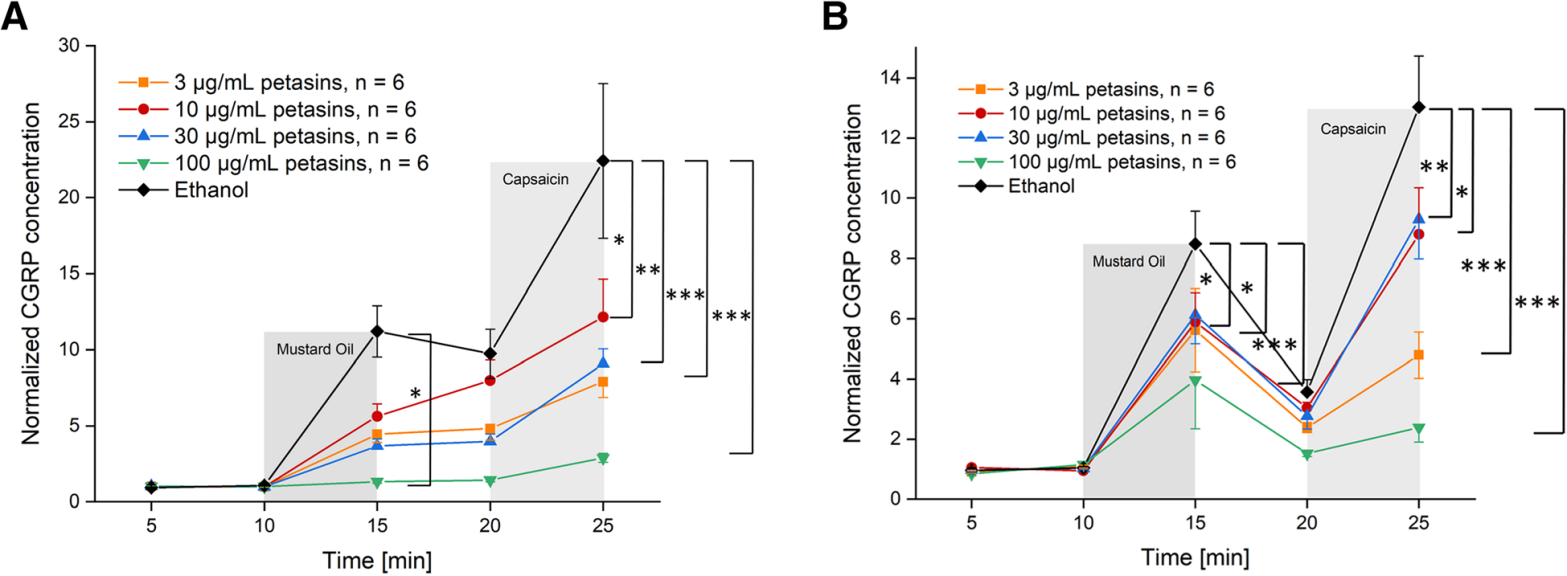
**a** Dura mater, rat. **b** Trigeminal ganglion, rat. Normalized CGRP release after pre-incubation with butterbur root extract (petasin concentrations 3, 10, 30, 100 μg/mL, *n* = 6 each) compared to pre-incubation with the vehicle ethanol (*n* = 12) for 1 h. Stimulation steps with 5 × 10^− 4^ M mustard oil (minutes 10–15 min) or 5 × 10^− 7^ M capsaicin (minutes 20–25); other steps with SIF incubation (minutes 5–10 and 15–20); *p < 0.05, **p < 0.01, ***p < 0.001

Stimulation with the TRPA1 receptor MO as well as with the TRPV1 receptor agonist Caps increased CGRP release significantly after ethanol incubation and after all concentrations of petasins but 100 μg/mL (Tables 1 and 2). However, a CGRP reducing effect of pre-incubation with butterbur root extract could be observed for all concentrations compared to the vehicle (ethanol), where the CGRP release after MO was 11.2-fold and after Caps 22.4-fold of the basal release. Nevertheless, there was no strict dose-dependent suppression of the CGRP release after 3, 10 and 30 μg/mL petasins (Fig. 1a), resulting in a MO-stimulated CGRP release of 4.5-, 5.6- and 3.7-fold, respectively, and a Caps-stimulated release of 7.9-fold, 12.2-fold and 9.1-fold, respectively, compared to the basal release. Significant differences between the serial samples (raw data, F_4,124_ = 104.82, *p* < 0.0001) and between the vehicle and the petasin doses (normalized data, F_4,31_ = 5.63, *p* = 0.0016) were indicated by two-way repeated measures ANOVA. LSD post hoc test revealed differences between the respective ethanol and petasin doses after stimulation steps (MO: 3 μg/mL: *p* = 0.098; 10 μg/mL: *p* = 0.169; 30 μg/mL: *p* = 0.067; 100 μg/mL: *p* = 0.018, LSD, *n* = 6 for each concentration; Caps: 3 μg/mL: *p* = 0.0009; 10 μg/mL: *p* = 0.015; 30 μg/mL: *p* = 0.002; 100 μg/mL: *p* < 0.0001; LSD, *n* = 6 for each concentration).

### Trigeminal ganglion, basal CGRP release

After 1 h incubation of trigeminal ganglia with different concentrations of the vehicle (SIF) containing ethanol (0.0316–1.055%), the CGRP concentration in the initial SIF solutions (basal release) varied between 23.2 and 49.3 pg/mL, which is mainly due to the inter-assay variation of the EIA kits. However, since there was no correlation of the basal release with the ethanol concentrations, likewise, there was no reason to assume that ethanol at these concentrations caused CGRP release itself. Therefore, the release values of all vehicle experiments (*n* = 12) were averaged and compared with the release values following 1 h treatment with different concentrations of petasins (3–100 μg/mL). There was also no evidence for a CGRP release stimulating effect of the petasins or isopetasin; basal values after SIF varied between 27.2 and 75.8 pg/mL without any correlation to the doses used. Raw data with deviations are displayed in Tables 4, 5, 6.

**Table 4:**
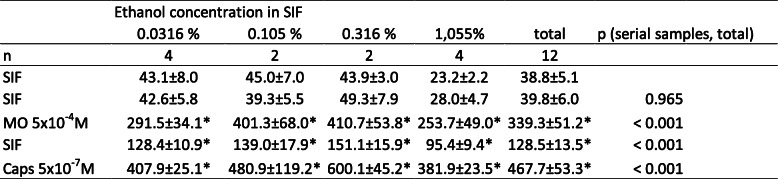
Trigeminal ganglia, different ethanol concentrations. CGRP release in pg/ml, all values are means ± SEM, *p < 0.05

**Table 5:**
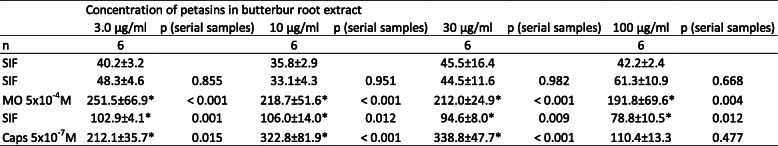
Trigeminal ganglia, different petasin concentrations. CGRP release in pg/ml, all values are means ± SEM, *p < 0.05

**Table 6:**
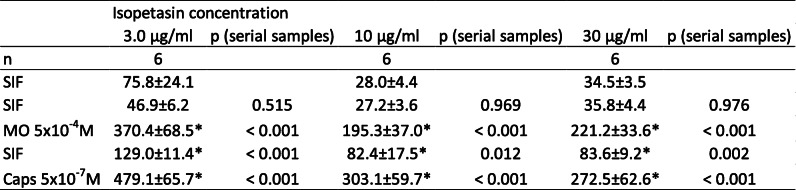
Trigeminal ganglia, different isopetasin concentrations. CGRP release in pg/ml, all values are means ± SEM, *p < 0.05

For all concentrations of petasins in butterbur root extract (3 μg/mL, 10 μg/mL, 30 μg/mL, 100 μg/mL) and the solvent ethanol without extract, the basal CGRP release from TG within 5 min was 41.5 ± 6.8 pg/mL (*n* = 36).

### Trigeminal ganglion, stimulated CGRP release

Stimulation with the TRPA1 receptor agonist MO increased CGRP release significantly for all concentrations of petasins. Pre-incubation with a concentration of 100 μg/mL petasins contained in butterbur root extract was the only concentration which was not followed by a significantly increased CGRP release after stimulation with the TRPV1 receptor agonist Caps (Tables 4 and 5). However, a CGRP-reducing effect of pre-incubation with butterbur root extract could be observed for all concentrations compared to the vehicle (ethanol), where the CGRP release after MO was 8.7-fold and after Caps 12.7-fold of the basal release. Nevertheless, there was no strict dose-dependent suppression of the CGRP release with almost similar normalized values after 3, 10 and 30 μg/mL petasins (Fig. 1b), resulting in a MO-stimulated CGRP release of 5.6-, 5.9- and 6.1-fold, respectively, and a Caps-stimulated CGRP release of 4.8-, 8.8- and 9.3-fold, respectively, compared to the basal release. Significant differences between the serial samples (raw data, F_4,124_ = 72.75, *p* < 0.0001) and between the vehicle and the petasin doses (normalized data, F_4,31_ = 10.26, *p* < 0.0001) were indicated by two-way repeated measures ANOVA. LSD post hoc test revealed differences between the respective ethanol and petasin doses after stimulation steps (MO: 3 μg/mL: *p* = 0.022; 10 μg/mL: *p* = 0.036; 30 μg/mL: *p* = 0.053; 100 μg/mL: *p* = 0.0008; LSD, *n* = 6 for each concentration; Caps: 3 μg/mL: *p* < 0.0001; 10 μg/mL: *p* = 0.005; 30 μg/mL: *p* = 0.012; 100 μg/mL: *p* < 0.0001; LSD, *n* = 6 for each concentration).

### Isopetasin, Wistar

The effect of pre-incubation with isopetasin solved in ethanol on the CGRP release was tested in the range of 3–30 μg/mL (3 μg/mL, 10 μg/mL, 30 μg/mL), and will later be compared to the effect of the same concentrations of petasins (3 μg/mL, 10 μg/mL, 30 μg/mL) included in the extract as described earlier. Raw data (see Tables 3, 6) was used for repeated measures analysis while normalized data was used for comparison between ethanol and isopetasin doses.

### Dura mater, basal CGRP release

After pre-incubation with all concentrations of isopetasin (3 μg/mL, 10 μg/mL, 30 μg/mL), the basal CGRP release from the dura mater encephali within 5 min was 16.9 ± 1.8 pg/mL (*n* = 18).

### Dura mater, stimulated CGRP release

Significant differences between the serial samples (raw data, F_4,104_ = 155.78 *p* < 0.0001), and between the vehicle and the isopetasin doses (F_3,26_ = 3.18, *p* = 0.04) were observed by two-way repeated measures ANOVA. Stimulation with the TRPA1 receptor agonist MO and the TRPV1 receptor agonist Caps increased CGRP release for all concentrations of isopetasin.

Even though most CGRP levels were elevated after stimulation steps, a reduction of the released CGRP compared to ethanol as well as significant differences between vehicle and 10 and 30 μg/mL isopetasin after stimulation with TRPV1 agonist capsaicin were observed (MO: 3 μg/mL: *p* = 0.193; 10 μg/mL: *p* = 0.085; 30 μg/mL: *p* = 0.133; LSD, *n* = 6 for each concentration; Caps: 3 μg/mL: *p* = 0.377; 10 μg/mL: *p* = 0.047; 30 μg/mL: *p* = 0.004; LSD, *n* = 6 for each concentration).

### Trigeminal ganglion, basal CGRP release

After pre-incubation with all concentrations of isopetasin (3 μg/mL, 10 μg/mL, 30 μg/mL), the basal CGRP release from trigeminal ganglia within 5 min was 41.4 ± 7.7 pg/mL (*n* = 18).

### Stimulated CGRP release

**Fig. 2:**
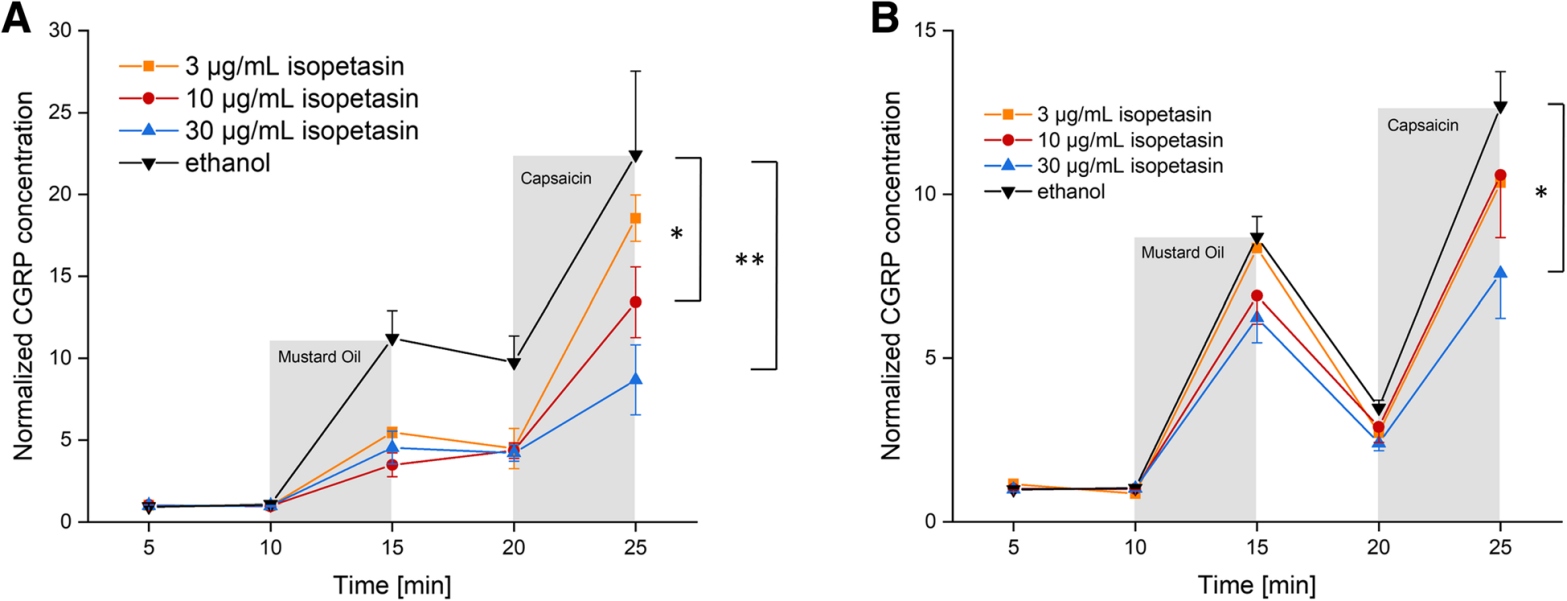
**a** Dura mater, rat. **b** Trigeminal ganglion, rat. Normalized CGRP release after pre-incubation with isopetasin concentrations (3, 10, 30 μg/mL, *n* = 6 each) compared to pre-incubation with ethanol (*n* = 12) for 1 h. Stimulation steps with 5 × 10^− 4^ M mustard oil (minutes 10–15) or 5 × 10^− 7^ M capsaicin (minutes 20–25); other steps with SIF incubation (minutes 5–10 and 15–20); *p < 0.05, **p < 0.01

Significant differences between the serial samples (F_4,100_ = 101.22 *p* < 0.0001) were revealed by two-way repeated measures ANOVA, while there was no significant effect between vehicle and the isopetasin doses (F_3,25_ = 1.41, *p* = 0.263). In detail, stimulation with the TRPA1 receptor agonist MO as well as the TRPV1 receptor agonist Caps increased CGRP for all concentrations of isopetasin (Fig. 2b).

Even though most CGRP levels were elevated after the stimulation steps, a slight reduction of the CGRP released from trigeminal ganglia compared to ethanol was observed for isopetasin. Furthermore, a significant reduction of CGRP release between ethanol and 30 μg/mL isopetasin after stimulation with TRPV1 agonist capsaicin was detected (MO: 3 μg/mL: *p* = 0.86; 10 μg/mL: *p* = 0.507; 30 μg/mL: *p* = 0.205; LSD, *n* = 6 for each concentration; Caps: 3 μg/mL: *p* = 0.227, *n* = 6; 10 μg/mL: *p* = 0.306, *n* = 5; 30 μg/mL: *p* = 0.01, *n* = 6; LSD).

### Effect of petasins in butterbur root extract compared to isopetasin

The effect of pre-incubation with petasins (3 μg/mL, 10 μg/mL, 30 μg/mL) contained in butterbur root extract and solved in ethanol on the CGRP release from dura mater encephali and trigeminal ganglia was compared to the effect of the same concentration of isopetasin (3 μg/mL, 10 μg/mL, 30 μg/mL) solved in ethanol on the basis of normalized data. Normalized data of the relative increase of CGRP after stimulation with MO or Caps are displayed in Tables 7 and 8.

**Table 7:**
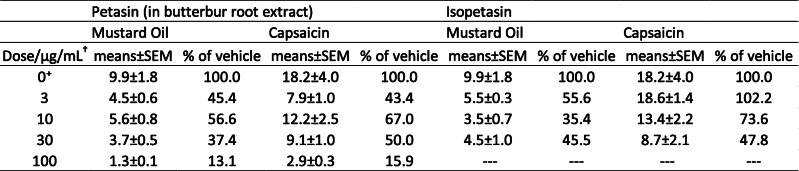
Dura mater encephali, Normalized data, Relative increase of CGRP after stimulation with Mustard Oil/Capsaicin. †dose of petasin/isopetasin, +vehicle (ethanol)

**Table 8:**
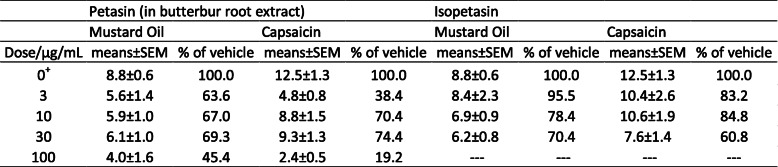
Trigeminal ganglia, Normalized data, Relative increase of CGRP after stimulation with Mustard Oil/Capsaicin. †dose of petasin/isopetasin, +vehicle (ethanol)

### Dura mater

Effects on the CGRP release from the dura mater encephali were compared after stimulating steps with MO and Caps. Univariate ANOVA did not indicate significant differences after stimulation with MO between the petasin and isopetasin group (normalized data, F_1,30_ = 0.03, *p* = 0.87) as well as the respective concentrations (normalized data, F_2,30_ = 0.64, *p* = 0.53). The LSD post hoc test revealed that stimulation with MO did not cause significant differences between the respective doses of the petasin and isopetasin group (3 μg/mL: *p* = 0.351; 10 μg/mL: *p* = 0.052; 30 μg/mL: *p* = 0.432; *n* = 6 for each concentration, LSD). However, for Caps stimulation, univariate ANOVA indicated significant differences between the petasin and isopetasin group (normalized data, F_1,30_ = 5.67, *p* = 0.02) but not between the respective concentrations (normalized data, F_2,30_ = 2.96, *p* = 0.07). According to LSD post hoc test, stimulation with Caps caused significant differences for the concentration 3 μg/mL (*p* = 0.0006, *n* = 6, LSD; 10 μg/mL: *p* = 0.656, *n* = 6, LSD; 30 μg/mL: *p* = 0.881, *n* = 6, LSD).

As mentioned above, the relative increase in CGRP release after stimulation with MO or Caps was not strictly dose-dependent for the pre-treatment with petasin extract and isopetasin, but both resulted in a decreased CGRP release compared to pre-preatment with the vehicle ethanol. The CGRP release after stimulation with MO ranged from approximately half (doses 3, 10 and 30 μg/mL) to about 15% for 100 μg/mL of petasins, similarly after stimulation with Caps (Table 7). CGRP levels after pre-treatment with isopetasin were in the same range except for the low dose of 3 μg/mL, which was not followed by a reduction in CGRP release upon stimulation with Caps.

Therefore, petasins and isopetasin seem to have almost similar quantitative effects on the reduction in CGRP release. The stronger effect after 3 μg/mL petasins may be due to other components contained in butterbur root extract.

### Trigeminal ganglion

Also for trigeminal ganglia, effects were compared after stimulating steps with MO and Caps. Univariate ANOVA did neither indicate significant differences after stimulation with MO nor with Caps between the petasin and isopetasin group (normalized data, MO: F_1,30_ = 1.17, *p* = 0.29; Caps: F_1,29_ = 1.59, *p* = 0.22) as well as between the respective concentrations (normalized data, MO: F_2,30_ = 0.17, *p* = 0.85; Caps: F_2,29_ = 0.67, *p* = 0.52). The LSD post hoc test revealed that stimulation with MO did not cause significant differences between the respective doses of the petasin and isopetasin group (3 μg/mL: *p* = 0.196; 10 μg/mL: *p* = 0.511; 30 μg/mL: *p* = 0.962; *n* = 6 for each concentration, LSD). However, stimulation with Caps caused significant differences for 3 μg/mL (*p* = 0.037, *n* = 6, LSD; 10 μg/mL: *p* = 0.507, *n* = 5, LSD; 30 μg/mL: *p* = 0.507, *n* = 6, LSD).

As mentioned above, in trigeminal ganglia the relative release of CGRP was again not strictly dose-dependent after previous incubation with both petasin extract and isopetasin. For the doses 10 and 30 μg/mL, CGRP release was in the same range with a reduction by one third, approximately, compared to vehicle. The lowest increase in CGRP release was again measured after a dose of 100 μg/mL of petasins resulting in a CGRP release of less than 50% after stimulation with MO and less than 20% after Caps, respectively, compared to vehicle. Interestingly, the reduction of CGRP released from trigeminal ganglia after pre-incubation with 3 μg/mL of isopetasin was lower than after pre-treatment with petasins in the extract (see Table 8).

In summary, in trigeminal ganglia, petasins and isopetasin seem to have almost similar quantitative effects on the CGRP release. The stronger effect after 3 μg/mL petasins may be due to other components contained in butterbur root extract.

### Butterbur root extract: effects in wild type, TRPV1−/− and TRPA1−/− mice

Wild type C57 BL/6 mice were compared to C57 BL/6 mice with functionally knocked out TRPA1 or TRPV1 receptors regarding the effect of pre-incubation with butterbur root extract on CGRP release. This effect was tested with a concentration of 30 μg/mL of petasins in butterbur root extract (solved in ethanol). Raw data with deviations are displayed in Tables 9, 10.

**Table 9:**
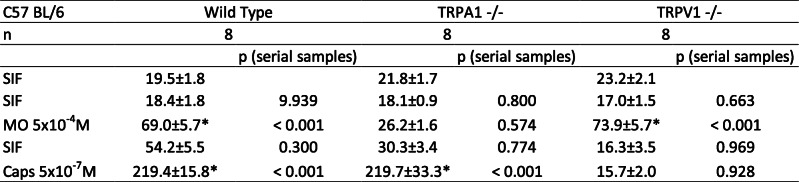
Dura mater encephali, CGRP release after pre-incubation with 30 μg/mL petasins in butterbur root extract. CGRP release in pg/mL, all values are means ± SEM, *p < 0.05

**Table 10:**
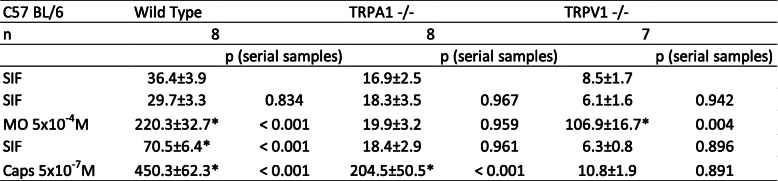
Trigeminal ganglia, CGRP release after pre-incubation with 30 μg/mL petasins in butterbur root extract. CGRP release in pg/mL, all values are means ± SEM, *p < 0.05

### Dura mater, basal CGRP release

For all C57 BL/6 mice (*n* = 24), the basal CGRP release from the dura mater encephali after two 5-min washing steps with SIF was 19.7 ± 1.6 pg/mL.

### Dura mater, stimulated CGRP release

**Fig. 3:**
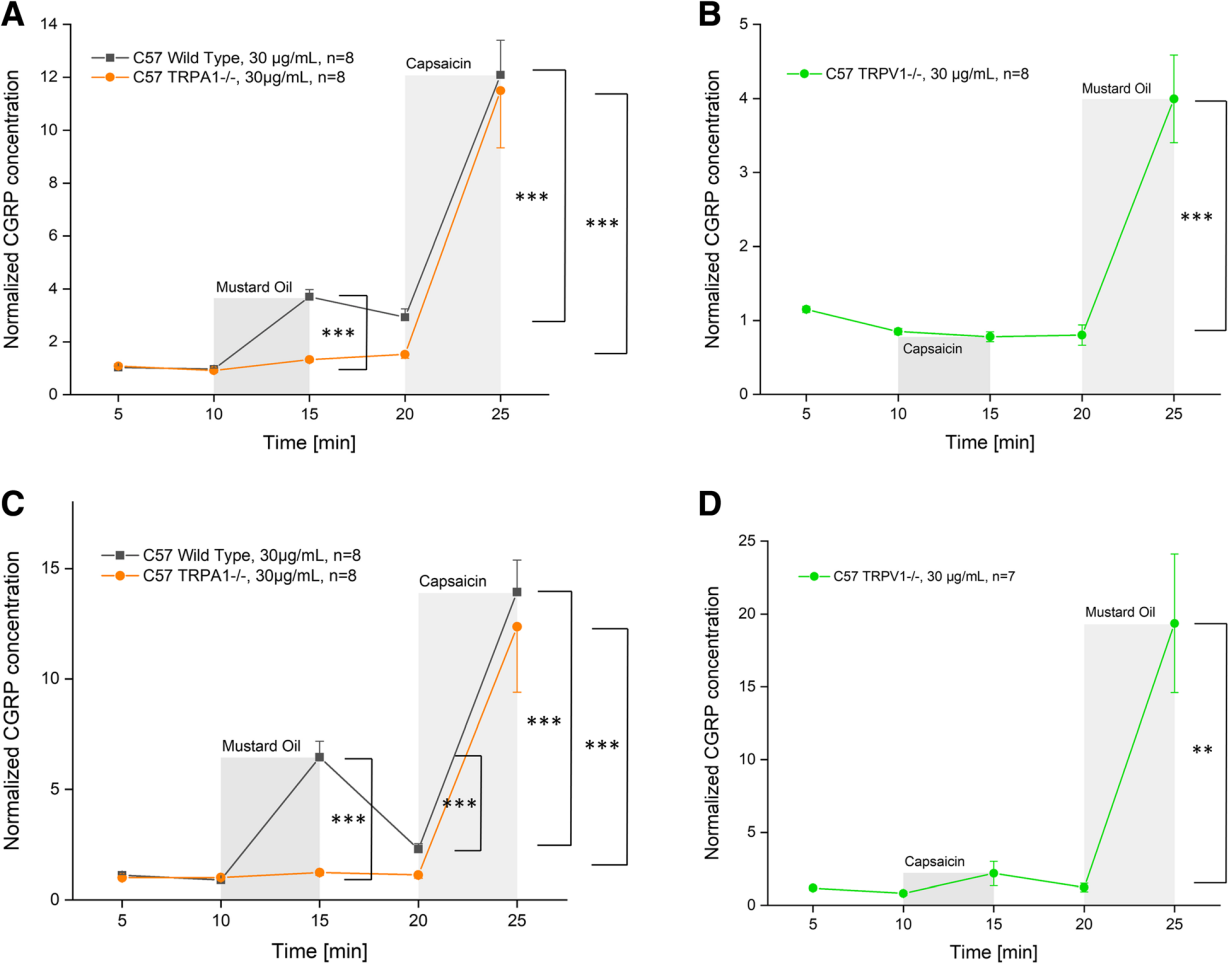
**a** Dura mater, C57 BL/6 wild-type and TRPA1 knocked out mouse. **b** Dura mater, TRPV1 knocked out mouse. **c** Trigeminal ganglion, C57 BL/6 wild-type and TRPA1 knocked out mouse. **d** Trigeminal ganglion, TRPV1 knocked out mouse. Normalized CGRP release after pre-incubation with butterbur root extract (petasin concentrations 30 μg/mL, *n* = 8 each; except for trigeminal ganglia of TRPV1 knocked out mice, *n* = 7) for 1 h. Stimulation steps with 5 × 10^− 4^ M mustard oil (**a** and **c**: minutes 10–15 for C57 BL/6 wild-type and TRPA1 knocked out mice, or minutes 20–25 for TRPV1 knocked out mice). Stimulation steps with 5 × 10^− 7^ M capsaicin (**b** and **d**: minutes 20–25 for C57 BL/6 wild type and TRPA1 knocked out mice, or minutes 10–15 for TRPV1 knocked out mice); other steps with SIF incubation (minutes 5–10 and 15–20); *p < 0.05, **p < 0.01, ***p < 0.001

Repeated measures ANOVA indicated significant differences after stimulation steps between the serial samples (raw data, F_4,84_ = 123.57, *p* < 0.0001). For wild type C57 BL/6, significantly increased CGRP rates could be recorded after stimulation steps with MO (3.7-fold) and Caps (12.1-fold) (MO: *p* = 0.0006, *n* = 8, LSD; Caps: *p* < 0.0001, *n* = 8, LSD). TRPA1 receptor knocked out mice did not show significantly elevated CGRP release (1.3-fold) after stimulation with MO (*p* = 0.574, *n* = 8, LSD), while the CGRP release after stimulation with Caps was increased to 11.5-fold (*p* < 0.0001, *n* = 8, LSD). The dura mater of TRPV1 receptor knocked out mice was first stimulated with the TRPV1 receptor agonist Caps resulting in a slightly decreased (0.8-fold) CGRP level (*p* = 0.929, *n* = 8, LSD). Stimulation with MO evoked a significantly increased CGRP release (4.0-fold) (*p* = 0.0001, *n* = 8, LSD) (Fig. 3a).

After stimulation steps, univariate ANOVA indicated significant differences between wild type, TRPA1 knocked out (ko) and TRPV1 ko receptor mice (normalized data, MO: F_2,21_ = 13.09, *p* = 0.0002; Caps: MO: F_2,21_ = 16.59, *p* < 0.0001). LSD post hoc test revealed significant differences between TRPA1 knocked out mice and the two other groups, respectively, after stimulation with MO (wild type: *p* = 0.0004, *n* = 8; TRPV1 ko: *p* = 0.0001, *n* = 8, LSD), as well as between TRPV1 knocked out mice and the two other groups after stimulation with Caps (wild type: *p* < 0.0001, *n* = 8; TRPA1 ko: *p* < 0.0001, *n* = 8, LSD) (Fig. 3b).

### Trigeminal ganglion, basal CGRP release

For all C57 BL/6 mice (*n* = 23), the basal CGRP release from trigeminal ganglia after two 5 min washing steps with SIF was 19.3 ± 2.8 pg/mL.

### Trigeminal ganglion, stimulated CGRP release

Repeated measures ANOVA indicated significant differences after stimulation steps between the serial samples (raw data, F_4,80_ = 55.62, *p* < 0.0001). For wild type C57 BL/6, significantly increased CGRP rates could be recorded after stimulation steps with MI (6.5-fold) and Caps (13.9-fold) (MO: *p* < 0.0001, *n* = 8, LSD; Caps: *p* < 0.0001, *n* = 8, LSD). TRPA1 receptor knocked out mice did not show significantly elevated CGRP release (1.2-fold) after stimulation with MO (*p* = 0.959, *n* = 8, LSD), while CGRP response after stimulation with Caps significantly (12.4-fold) increased (*p* < 0.0001, *n* = 8, LSD). TG of TRPV1 receptor knocked out mice were first stimulated with the TRPV1 receptor agonist Caps resulting in a slightly increased CGRP level (2.2-fold) (*p* = 0.891, *n* = 7, LSD). Stimulation with MO was followed by significantly elevated CGRP release (19.4-fold) (*p* = 0.004, *n* = 7, LSD) (Fig. 3c).

After stimulation steps, univariate ANOVA indicated significant differences between wild type, TRPA1 knocked out and TRPV1 knocked out receptor mice (normalized data, MO: F_2,18_ = 10.09, *p* = 0.001; Caps: F_2,18_ = 5.46, *p* = 0.01). LSD post hoc test revealed significant differences between TRPV1 knocked out mice and the two other groups, respectively, after stimulation with MO (wild type: *p* = 0.008, *n* = 8; TRPA1 ko: *p* = 0.0003, *n* = 8, LSD), as well as between TRPV1 knocked out mice and the two other groups after stimulation with Caps (wild type: *p* = 0.048, *n* = 8; TRPA1 ko: *p* = 0.004, *n* = 8; LSD) (Fig. 3d).

## Discussion

Our results suggest that petasins in butterbur root extract as well as isopetasin have an inhibiting effect on the TRPV1- and TRPA1-mediated CGRP release. After pre-treatment with petasins, CGRP released from the dura mater and trigeminal ganglia was – partly significantly – reduced after stimulation with MO or Caps compared to pre-incubation with ethanol as vehicle. Pre-treatment with 100 μg/mL of petasins in butterbur root extract resulted in a non-significant CGRP release from both the dura mater and trigeminal ganglia compared with previous incubation steps.

For TRPA1 receptor channels, Benemei et al. [32] already reported a reduction of CGRP release from mouse dorsal spinal cord slices upon stimulation with MO after 30 min exposure to a high concentration of isopetasin (300 μM). We confirmed these findings in peripheral tissues, the dura mater and trigeminal ganglia, even after treatment with lower concentrations of petasins. However, different to the findings of Benemei et al., who did not see an activation of TRPV1 channels expressed in HEK293 cells by isopetasin, we observed also a decrease in CGRP release upon stimulation of TRPV1 following pretreatment with petasins. The stimulating agent Caps is a specific and direct agonist of TRPV1 [14, 47] and, as we showed a significant reduction of the Caps-induced TRPV1 response, it is more than evident that TRPV1 is another site of action of petasins. Since it has been shown that TRPA1 and TRPV1 channels interact functionally undergoing reciprocal sensitization [48, 49], it seems possible that TRPV1 requires the presence of TRPA1 to respond effectively to petasins, which may be worth to be explored in further experiments. Immunohistochemically, TRPV1 receptor channels are found co-localized with CGRP-positive neurons [10, 11] which are probably of immense importance for the transduction and amplification of pain in the trigeminovascular system [50–52]. TRPV1 receptor channels are often co-expressed with TRPA1 receptor channels [12–14], and therefore, it is likely that both channels are affected simultaneously by petasins and act together in the trigeminovascular system.

Although there is no strict dose-dependency, we demonstrated that the effects of petasins generally increased at higher doses. Pre-treatment with 100 μg/mL petasin in the extract resulted in the most effective suppression of CGRP release from the dura mater and trigeminal ganglia. After stimulation with MO and Caps, CGRP was not significantly increased compared to previous incubation steps. In trend, effects of petasin in the extract and pure isopetasin were similar, however, isopetasin doses were investigated up to 30 μg/mL only. Therefore, further analysis of doses might be interesting: A dose of 100 μg/mL of isopetasin in comparison to 100 μg/mL of petasins in the extract as well as an additional dose testing in the range of > 30 μg/mL and < 100 μg/mL would probably allow more exact statements about the effectiveness of the respective doses.

Interestingly, after pre-treatment with 3 μg/mL of petasins in the extract or isopetasin and stimulation with Caps, TRPV1 receptor channel activity was significantly different between the two substances. CGRP release after pre-incubation with 3 μg/mL of isopetasin and stimulation with Caps was significantly higher than after the same dose of petasins in butterbur root extract. This phenomenon is reported for this lowest concentration and after stimulation with Caps only. It is not very probable that the effect is based on different actions of petasin and isopetasin, as petasin converts into isopetasin spontaneously [32]. The difference is possibly due to other components contained in the extract, which might be without effect at higher doses of petasins. Possibly antinociceptive effects of TRPA1 receptor channels are more active after pre-treatment with petasins in butterbur root extract than after pure isopetasin. The effect of other components of butterbur root extract and their impact on TRP receptor channels might be an interesting subject of further investigation.

The experiments with functionally deleted TRP receptors have been controlled by wild type receptor configuration with pre-treatment of the same concentration of petasins in the extract. However, a control without pre-incubation with an active substance did not take place, and thus a comparison without pre-treatment is lacking. Nevertheless, our data suggest that the effect indeed exists according to the earlier dose finding experiments, in which we not only compared different doses but also controlled the effects by a pre-incubation with ethanol only. It is not surprising that the results of the experiments with functionally deleted TRP receptors indicate that receptor responses take place neither in the dura nor in TG of the animals with the respective functionally deleted TRP receptor channels. Nevertheless, the quantitative CGRP release after stimulation of TRPA1 receptors channels of TRPV1−/− mice compared with the CGRP release after stimulation of TRPA1 of wild type mice was almost identical in the dura. As we observed the same effect for TRPV1 receptor channels in TRPA1−/− mice compared to wild type, a cooperative effect between the two TRP receptor channels is not evident. Adding to the discussion above, this might be due to a lack of functional synergism of these receptors in the dura possibly abolished by pre-treatment with petasins. Likewise, in trigeminal ganglia of TRPA1−/− mice compared to wild type, quantitative CGRP release after stimulation with Caps was in the same range, so that we suggest an inhibited synergism of the receptors there, too. However, the TRPA1 receptor response from trigeminal ganglia with functionally deleted TRPV1 receptor channels was three times higher (19.4-fold) in comparison to wild type (6.5-fold). Benemei et al. [32] suggest that isopetasin at lower concentrations (100 μM) may act as a partial agonist of TRPA1 receptor channels resulting in an isopetasin-evoked CGRP release in TRPV1-positive neurons. Since CGRP release is calcium-dependent, and Ca^2+^-influx mediated by TRPV1 is not possible via these channels in TRPV1−/− mice, the influx is possibly solely mediated by activated TRPA1 receptor channels being permeable for calcium cations, too [14]. Thus, in trigeminal ganglia, a co-expression of TRP receptors may be of functional importance in an inverse way. A possible anti-nociceptive synergistic action after pre-treatment with petasins may be effective only if both, TRPV1 and TRPA1, receptor channels are intact. It might be that the main site of action of petasins in sensory neurons of trigeminal ganglia are TRPV1 receptor channels, and that TRPA1 effects are modulated by TRPV1, as discussed [48, 49]. Consequently, TRPV1 knocked out receptor channels might result in a pro-nociceptive effect of the still intact TRPA1 receptor channels, which have not been inactivated by petasins. The idea of an increased TRPA1 receptor response after treatment with petasins due to functionally deleted TRPV1 receptor channels is contradictory to previous findings of CGRP release experiments, in which stimulation with MO resulted in a lower TRPA1 response in TRPV1−/− mice [15]. However, this discrepancy might be again due to pre-treatment with petasins.

As mentioned above, butterbur root extract is successfully used in migraine prophylaxis [20–22]. However, long-term effects of either petasins in the extract or pure isopetasin could not be taken into consideration in this work. The pre-incubation period lasted 1 h so that only a snapshot of possible effects has been made, and we must assume that a repeated long-term application leads to higher and stronger results. On the other hand, there may also be other components contained in the extract that may be effective after a regular and repeated exposition. As the structures of the ex vivo experiments performed in this work cannot be held viable over a longer period, a partly different method should be taken into consideration. In order to achieve an understanding of long-term effects and mechanisms, repeated in vivo application of petasins in the extract and isopetasin followed by an examination of the in vitro CGRP release in comparison to placebo or nocebo might be an interesting approach.

An issue, which could not be examined in the experimental rodent preparation, is the fact that the plant butterbur contains pyrrolizidines alkaloids (PAs) which are associated with hepatotoxicity [34, 53]. The PAs of the plant’s rhizome are non-toxic hydrophile N-oxides, however, they are reduced by gut bacteria and subsequently resorbed as lipophile PAs. In the liver, oxidation via cytochrome P450 and further decomposition results in toxic and carcinogen PAs which may cause liver tumors [34, 54]. Therefore, it is important to remove the toxic constituents and to use a PA-free butterbur root extract for migraine prevention [39, 40].

## Conclusion

In summary, this work shows that butterbur root extract with its active agents petasin and isopetasin possibly not only affects TRPA1 receptor channels but also TRPV1 resulting in decreased CGRP levels in a well-established ex vivo model. However, several ambiguities derive from the current findings: Future approaches could handle the question, if TRPA1 and TRPV1 are the only receptors involved in the site of action of petasins, and if there is an additional contribution of e.g. transient receptor potential melastatin type 8 (TRPM8) receptor channels. Furthermore, it is of considerable interest, which of the receptor channels is the main actor, and how cooperative effects within the TRP family mediate the action. Another subject are possible other active components of the extract that are of therapeutic relevance, and how a long-term treatment influences the results.

## Data Availability

All data generated and analysed during this study are included in this article.

## Abbreviations

AChE: Acetylcholinesterase
Caps: Capsaicin
CGRP: Calcitonin gene-related peptide
ko: Knocked out
MO: Mustard oil
PAs: Pyrrolizidines alkaloids
SIF: Synthetic interstitial fluid
TG: Trigeminal ganglion
TRPA1: Transient receptor potential ankyrin type 1
TRPM8: Transient receptor potential melastatin type 8
TRPV1: Transient receptor potential vanilloid type 1
VGCC: Voltage-gated Ca^2+^-channel

## References

[1] MacGregor EA (2017). Migraine. Ann Intern Med.

[2] Dodick DW (2018). Migraine. Lancet.

[3] Silberstein SD (2004). Migraine. Lancet.

[4] Goadsby PJ, Holland PR, Martins-Oliveira M, Hoffmann J, Schankin C, Akerman S (2017). Pathophysiology of migraine: a disorder of sensory processing. Physiol Rev.

[5] Goadsby PJ, Holland PR (2019). An update: pathophysiology of migraine. Neurol Clin.

[6] Noseda R, Burstein R (2013). Migraine pathophysiology: anatomy of the trigeminovascular pathway and associated neurological symptoms, cortical spreading depression, sensitization, and modulation of pain. Pain.

[7] Burstein R, Noseda R, Borsook D (2015). Migraine: multiple processes, complex pathophysiology. J Neurosci.

[8] Ashina M, Hansen J, Do T (2019). Migraine and the trigeminovascular system-40 years and counting. Lancet Neurol.

[9] Iyengar S, Johnson KW, Ossipov MH, Aurora SK (2019). CGRP and the trigeminal system in migraine. Headache.

[10] Price TJ, Flores CM (2007). Critical evaluation of the colocalization between calcitonin gene-related peptide, substance P, transient receptor potential vanilloid subfamily type 1 immunoreactivities, and isolectin B4 binding in primary afferent neurons of the rat and mouse. J Pain.

[11] Dux M, Rosta J, Messlinger K (2020). TRP channels in the focus of trigeminal nociceptor sensitization contributing to primary headaches. Int J Mol Sci.

[12] Bautista D, Jordt S, Nikai T (2006). TRPA1 mediates the inflammatory actions of environmental irritants and proalgesic agents. Cell.

[13] Bautista D, Pellegrino M, Tsunzaki M (2013). TRPA1: a gatekeeper for inflammation. Annu Rev Physiol.

[14] Julius D (2013). TRP channels and pain. Annu Rev Cell Dev Biol.

[15] Denner AC, Vogler B, Messlinger K, de Col R (2017). Role of transient receptor potential ankyrin 1 receptors in rodent models of meningeal nociception – experiments in vitro. Eur J Pain.

[16] Fischer MJ, Balasuriya D, Jeggle P (2014). Direct evidence for functional TRPV1/TRPA1 heteromers. Pflugers Arch.

[17] Andersson DA, Gentry C, Alenmyr L, Killander D, Lewis SE, Andersson A, Bucher B, Galzi JL, Sterner O, Bevan S, Högestätt ED, Zygmunt PM (2011). TRPA1 mediates spinal antinociception induced by acetaminophen and the cannabinoid Δ (9)-tetrahydrocannabiorcol. Nat Commun.

[18] Teicher C, De Col R, Messlinger K (2017). Hydrogen sulfide mediating both excitatory and inhibitory effects in a rat model of meningeal nociception and headache generation. Front Neurol.

[19] Edmeads J (1999). History of migraine treatment. Can J Clin Pharmacol.

[20] Grossmann W, Schmidramsl H (2001). An extract of Petasites hybridus is effective in the prophylaxis of migraine. Altern Med Rev.

[21] Diener HC, Rahlfs VW, Danesch U (2004). The first placebo-controlled trial of a special butterbur root extract for the prevention of migraine: reanalysis of efficacy criteria. Eur Neurol.

[22] Lipton RB, Göbel H, Einhäupl KM (2004). Petasites hybridus root (butterbur) is an effective preventive treatment for migraine. Neurology.

[23] Messlinger K, Hanesch U, Baumgärtel M (1993). Innervation of the dura mater encephali of cat and rat: ultrastructure and calcitonin gene-related peptide-like and substance P-like immunoreactivity. Anat Embryol.

[24] Edvinsson L, Ekman R, Jansen I, McCulloch J, Uddman R (1987). Calcitonin gene-related peptide and cerebral blood vessels: distribution and vasomotor effects. J Cereb Blood Flow Metab.

[25] Lennerz JK, Rühle V, Ceppa EP, Neuhuber WL, Bunnett NW, Grady EF, Messlinger K (2008). Calcitonin receptor-like receptor (CLR), receptor activity-modifying protein 1 (RAMP1), and calcitonin gene-related peptide (CGRP) immunoreactivity in the rat trigeminovascular system: differences between peripheral and central CGRP receptor distribution. J Comp Neurol.

[26] Eftekhari S, Salvatore C, Calamari A (2010). Differential distribution of calcitonin gene-related peptide and its receptor components in the human trigeminal ganglion. Neuroscience.

[27] Eftekhari S, Edvinsson L (2011). Calcitonin gene-related peptide (CGRP) and its receptor components in human and rat spinal trigeminal nucleus and spinal cord at C1-level. BMC Neurosci.

[28] Zaidi M, Breimer LH, MacIntyre I (1987). Biology of peptides from the calcitonin genes. Q J Exp Physiol.

[29] Lundberg J, Franco-Cereceda A, Alving K (1992). Release of calcitonin gene-related peptide from sensory neurons. Ann N Y Acad Sci.

[30] Edvinsson L, Haanes KA, Warfvinge K, Krause DN (2018). CGRP as the target of new migraine therapies — successful translation from bench to clinic. Nat Rev Neurol.

[31] Messlinger K (2018). The big CGRP flood - sources, sinks and signalling sites in the trigeminovascular system. J Headache Pain.

[32] Benemei S, De Logu F, Li Puma S (2017). The anti-migraine component of butterbur extracts, isopetasin, desensitizes peptidergic nociceptors by acting on TRPA1 cation channel. Br J Pharmacol.

[33] Slavin M, Bourguignon J, Jackson K, Orciga MA (2016). Impact of food components on in vitro calcitonin gene-related peptide secretion-a potential mechanism for dietary influence on migraine. Nutrients.

[34] Kälin P (2003). Gemeine pestwurz (petasites hybridus) – portrait einer arzneipflanze. Complement Med Res.

[35] Bucher K (1951). Über ein antispastisches prinzip in petasites officinals moench. Arch Exper Path u Pharmakol.

[36] Bauer H, Kuehne P (1986). Therapie von Harnleiterkoliken mit einem neuen Spasmoanalgetikum. Therapiewoche.

[37] Schapowal A (2002). Randomised controlled trial of butterbur and cetirizine for treating seasonal allergic rhinitis. BMJ.

[38] Schapowal A (2005). Treating intermittent allergic rhinitis: a prospective, randomized, placebo and antihistamine-controlled study of butterbur extract Ze 339. Phytother Res.

[39] Aydin A, Zerbes V, Parlar H (2013). The medical plant butterbur (Petasites): a analytical and physiological (re)view. J Pharm Biomed Anal.

[40] Danesch U, Rittinghausen R (2003). Safety of a patented special butterbur root extract for migraine prevention. Headache.

[41] Thomet OAR, Wiesmann UN, Blaser K, Simon HU (2001). Differential inhibition of inflammatory effector functions by petasin, isopetasin and neopetasin in human eosinophils. Clin Exp Allergy.

[42] Wang GJ, Shum AY, Lin YL, Liao JF, Wu XC, Ren J, Chen CF (2001). Calcium channel blockade in vascular smooth muscle cells: major hypotensive mechanism of S-petasin, a hypotensive sesquiterpene from Petasites formosanus. J Pharmacol Exp Ther.

[43] Wang GJ, Wu XC, Lin YL, Ren J, Shum AYC, Wu YY, Chen CF (2002). Ca2+ channel blocking effect of iso-S-petasin in rat aortic smooth muscle cells. Eur J Pharmacol.

[44] Wang GJ, Liao JF, Hintz KK, Shi CC, Chen CF, Chen WP, Su MJ, Lin YL, Ren J (2004). Calcium-antagonizing activity of S-petasin, a hypotensive sesquiterpene from Petasites formosanus, on inotropic and chronotropic responses in isolated rat atria and cardiac myocytes. Naunyn Schmiedeberg's Arch Pharmacol.

[45] Horak S, Koschak A, Stuppner H (2009). Use-dependent block of voltage-gated Cav2.1 Ca2+ channels by petasins and eudesmol isomers. J Pharmacol Exp Ther.

[46] Ko WC, Lei CB, Lin YL, Chen CF (2001). Mechanisms of relaxant action of S-petasin and S-isopetasin, sesquiterpenes of petasites formosanus, in isolated guinea pig trachea. Planta Med.

[47] Patapoutian A, Tate S, Woolf CJ (2009). Transient receptor potential channels: targeting pain at the source. Nat Rev Drug Discov.

[48] Salas MM, Hargreaves KM, Akopian AN (2009). TRPA1-mediated responses in trigeminal sensory neurons: interaction between TRPA1 and TRPV1. Eur J Neurosci.

[49] Patil MJ, Salas M, Bialuhin S, Boyd JT, Jeske NA, Akopian AN (2020). Sensitization of small-diameter sensory neurons is controlled by TRPV1 and TRPA1 association. FASEB J.

[50] Fernandes E, Fernandes M, Keeble J (2012). The functions of TRPA1 and TRPV1: moving away from sensory nerves. Br J Pharmacol.

[51] Benemei S, Fusi C, Trevisan G, Geppetti P (2014). The TRPA1 channel in migraine mechanism and treatment. Br J Pharmacol.

[52] Dussor G, Cao Y-Q (2016). TRPM8 and migraine. Headache.

[53] Mauskop A (2013). Evidence-based guideline update: NSAIDs and other complementary treatments for episodic migraine prevention in adults: report of the Quality Standards Subcommittee of the American Academy of Neurology and the American Headache Society. Neurology.

[54] Habs M, Habs H, Forth W (1991). Kanzerogene naturprodukte: risikobewertung pyrrolizidinhaltiger arzneistoffe. Dtsch Arztebl.

